# Low Work-function Poly(3,4-ethylenedioxylenethiophene): Poly(styrene sulfonate) as Electron-transport Layer for High-efficient and Stable Polymer Solar Cells

**DOI:** 10.1038/srep12839

**Published:** 2015-08-04

**Authors:** Yong Zhang, Lie Chen, Xiaotian Hu, Lin Zhang, Yiwang Chen

**Affiliations:** 1 College of Chemistry/Institute of Polymers, Nanchang University, 999 Xuefu Avenue, Nanchang 330031, China; 2 Jiangxi Provincial Key Laboratory of New Energy Chemistry, Nanchang University, 999 Xuefu Avenue, Nanchang 330031, China

## Abstract

Low-work-function poly(3,4-ethylenedioxythiophene):poly(styrene sulfonate) (PEDOT:PSS) modified with polyethylenimine (PEIE) was used as an electron transport layer (ETL) for polymer solar cells (PSCs). A thin layer of PEIE film was spin-coated onto the surface on the PEDOT:PSS films, thus substantially changing their charge selectivity from supporting hole transport to supporting electron transport. It was also found that the PEDOT:PSS/PEIE ETL exhibited higher interfacial contact, a more favorable active morphology, and improved charge mobility. By virtue of these beneficial properties, inverted PSCs based on low-bandgap semiconducting photoactive layers achieved a notably improved power conversion efficiency (PCE) of 7.94%, superior even to the corresponding performance of devices with only a ZnO layer. Surpassing our expectations, compared with the extreme degradation of device stability observed when pure PEDOT:PSS is used, PEIE-modified PEDOT:PSS can considerably suppress device degradation because of the hydrophobic and alkaline nature of PEIE, which not only reduces the hygroscopicity of the PEDOT:PSS but also neutralizes the acidic PEDOT:PSS and thus prevents the corrosion of the ITO cathode. These results demonstrate the potential of PEIE-modified PEDOT:PSS for use as an efficient ETL in commercial printed electronic devices.

Polymer solar cells (PSCs) are attracting interest as potential sources of renewable and clean energy because of their attractive advantages of low-cost large-areas fabrication on light weight flexible substrates^1^,^2^,^3^,^4^,^5^. In the last few years, considerable efforts have been made to improve PSC performance, and devices based on bulk heterojunction (BHJ) structures have been reported to exhibit power conversion efficiencies of up to 10%, thus providing impetus for their successful commercialization^6^,^7^,^8^. Despite the recent achievements in cell efficiency, device stability remains a crucial challenge for commercialized PSCs. In general, BHJ-based conventional-structure PSCs consist of an active layer and two charge-collecting layers that are sandwiched between an indium-tin-oxide (ITO) anode and a low-work-function metal cathode (e.g., Al). Usually, poly(3,4-ethylenedioxythiophene):poly(styrene sulfonate) (PEDOT:PSS) is the most commonly used hole transport layer (HTL) material for ITO modification because of its high, stable work function (WF)^9^. A PEDOT:PSS interfacial layer also yields a high-performance PSC with improved ohmic contact with the active layer, enhanced hole collection^10^,^11^, an increased open-circuit voltage (*V*_OC_)^11^, and improved areal electrical uniformity in a complete PSC^12^. However, the conventional structure suffers from the cell degradation caused by the diffusion of oxygen into the active layer through pinholes and the corrosion of the ITO by the acidic and hygroscopic PEDOT:PSS^13^,^14^,^15^.

To circumvent this device degradation, an inverted structure has been introduced in which the charge collection behavior of the electrodes is opposite to that of the conventional structure. In the inverted structure, an air-stable high-work-function metal (such as Ag or Au) is used as the anode to collect holes, whereas the ITO acts as the cathode to collect electrons. Stability is improved in the inverted structure because the air-stable high-work-function metal electrode serves to self-encapsulate the cell and an ITO/PEDOT:PSS interface is avoided. For the efficient transport of electrons to the ITO, inorganic metal oxides, such as solution-processable (via a sol-gel precursor and nanoparticle solutions) zinc oxide (ZnO) or titanium oxide (TiO_x_), are the materials that are most widely used as electron transport layers (ETLs) between the ITO and the active layer^16^,^17^,^18^. However, to achieve better crystallinity of the metal oxides to ensure high charge-carrier mobility, the conversion of the precursor to a metal oxide requires a high-temperature (over 200 °C) annealing process, which limits the printable applications of this approach^19^,^20^. To avoid the inherent disadvantages of using inorganic materials as ETLs, organic interlayer materials, such as fullerene derivatives^21^,^22^, self-assembled monolayers^23^ and conjugated polyelectrolytes (CPEs)^24^,^25^,^26^, have been employed to efficiently transport electrons from the ITO; however, most such materials require tedious synthesis.

If PEDOT:PSS, a commercial and ubiquitous HTL material, can also serve as an ETL^27^ while simultaneously maintaining its stability on an ITO substrate, it could be compatible with the printing process for practical flexible-substrate devices. It has been reported that the addition of 80% ethoxylated polyethylenimine (PEIE), an organic polymer, can dramatically reduce the WF of PEDOT:PSS and alter its charge selectivity to modify it from an HTL into a transparent cathode^28^,^29^,^30^. Inspired by these findings, in this study, a thin layer of PEIE film (~10 nm) is spin-coated onto the surfaces of PEDOT:PSS films to substantially change their charge selectivity from supporting hole transport to supporting electron transport. In this approach, a tunable WF can be readily attained by simply controlling the pH value of the PEIE solution. It should be noted that Zhou *et al.*^28^ only change the WF of ITO by adding the HPF6 or NaOH in PEIE, but do not discover the application of the tunable WF for the traditional hole-transporting PEDOT:PSS. In our work, we further explain the tunable WF PEDOT:PSS as an electron-transport layer for inverted polymer solar cells. After the PEDOT:PSS is coated with a layer of PEIE, the interfacial contact with the active layer, electron transport and light absorption are found to be greatly improved. As a result, this novel solution-processed PEDOT:PSS/PEIE ETL exhibits power conversion efficiency (PCE) values of 3.64% and 7.94% in inverted PSCs based on poly(3-hexylthiophene):[6,6]-phenyl C61-butyric acid methyl ester (P3HT:PC_61_BM) and on the low-bandgap semiconducting polymer poly{4,8-bis[(2-ethylhexyl)oxy]benzo[1,2-b:4,5-b’]dithiophene-2,6-diyl-alt-3-fluoro-2-[(2-ethylhexyl)carbonyl]thieno[3]thiophene-4,6-diyl}:[6,6]-phenyl C71-butyric acid methyl ester (PTB7:PC_71_BM)^6^,^31^,^32^, respectively. Notably, these values are even higher than those for devices with bare ZnO as the ETL fabricated under the same conditions. Surpassing our expectations, compared with the extreme degradation in device stability observed when pure PEDOT:PSS is used, PEIE-modified PEDOT:PSS can considerably stabilize device performance. We find that the remarkably improved long-term stability provided by the hydrophobic and alkaline PEIE not only reduces the hygroscopicity of PEDOT:PSS but also neutralizes the acidic PEDOT:PSS to prevent the corrosion of the ITO.

## Results

### Properties of modified PEDOT:PSS films

**Figure 1:**
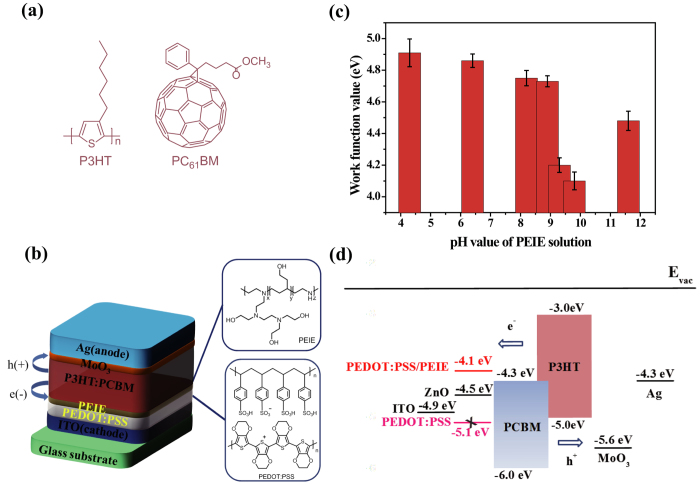
Solar cell structure and the molecular structures and work function histogram of the materials used in the cells. (**a**) Structures of P3HT and PC_61_BM. (**b**) The cell structure of the P3HT:PC_61_BM solar cell. The inset shows the molecular structures of PEDOT:PSS and PEIE. (**c**) Work function histogram of PEDOT:PSS modified with PEIE prepared from a 2-methoxyethanol solution (pH = 9.8), PEIE solutions with HPF_6_ added (pH = 4.3 **–** 9.3) and a PEIE solution with NaOH added (pH = 11.6). (**d**) Energy-level diagram of the cell structure.

The molecular structures of P3HT, PC_61_BM, PEDOT:PSS and PEIE as well as the cell structure and the energy levels of the components used in the cells are shown in Fig. 1a,b,d, respectively. The work function of PEDOT:PSS was characterized via ultraviolet photoelectron spectroscopy (UPS) and found to be approximately 5.1 eV (see Supplementary Fig. S1 online), which is much higher than the lowest unoccupied molecular orbital (LUMO) of PC_61_BM (4.3 eV). As a result, hole transport to ITO is favorable but electron transport is not (Fig. 1d). It has been reported that PEIE can act as a surface modifier to effectively reduce the WF of a conductor or semiconductor; therefore, PEIE was deposited onto the surface of PEDOT:PSS to alter its behavior from that of a hole transport layer to behavior suitable for electron extraction. To precisely control the WF of the PEDOT:PSS, a series of ultrathin PEIE films (approximately 10 nm in thickness) with different pH values (4.3, 6.4, 8.2, 8.9, 9.3, 9.8, and 11.6, achieved by adding either hexafluorophosphoric acid (HPF_6_) or NaOH^28^) was spin-coated onto the surfaces of PEDOT:PSS films. As expected, the WFs of the PEDOT:PSS/PEIE films could be tuned from 4.1 to 4.9 eV by modifying the pH value of the original PEIE solution (Fig. 1c and Supplementary Fig. S1 online). The interaction between the PEDOT:PSS and the PEIE could be clearly characterized based on the XPS spectra of the PEDOT:PSS/PEIE (see Supplementary Fig. S2 online); it was found that differences in the solution pH value primarily affected the degree of protonation of the amine groups in the PEIE. When the PEIE layers were processed from HPF_6_ solutions, these samples exhibited an obvious N1s peak at 402.2 eV, indicative of protonated amines. However, the PEIE layers that were prepared from the more basic solutions exhibited a high ratio of neutral [N] to protonated amines at 399.5 eV and a distinct downward shift in the WF, as revealed in the XPS and UPS spectra. Therefore, the neutral amine groups in PEIE are primarily responsible for the substantial changes observed in the WF through the creation of an interfacial dipole moment^28^. Among these PEDOT:PSS films modified with PEIE prepared at different pH values, those prepared using the pH = 9.8 solution exhibited the lowest WF of ~4.1 eV with a vacuum level shift of ~1.0 eV, which is even lower than that of commonly used ZnO ETLs (4.5 eV)^28^. This reduced WF is well matched to the LUMO levels of the fullerene acceptor. Hence, the PEDOT:PSS was successfully modified from an HTL to an ETL, thereby enabling electron transport from the acceptor to the ITO through the PEDOT:PSS/PEIE. Moreover, the presence of PEIE does not destroy the transparency of PEDOT:PSS but rather enhances its transmission at 400 nm to over 85% for improved light harvesting (see Supplementary Fig. S3 online).

**Figure 2:**
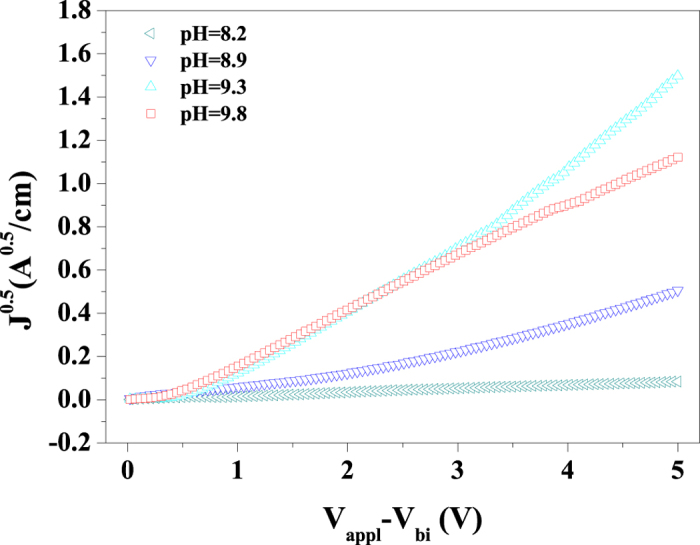
Space-charge-limited current (SCLC) mobility measurements of various interfacial layers. *J*^0.5^–*V* characteristics of electron-only devices with PEDOT:PSS/PEIE cathode interfacial layers fabricated from solutions of different pH values.

The electron transport capabilities of the PEDOT:PSS/PEIE interfacial layers prepared from solutions of different pH values were evaluated in electron-only devices with a configuration of ITO/interlayer/P3HT:PC_61_BM/LiF/Al. The *J*^0.5^−*V* curves of the electron-only devices were measured using the space-charge-limited current (SCLC) model in accordance with the Mott−Gurney equation (details of the mobility measurements are provided in the methods)^33^,^34^,^35^,^36^. The x-axes applied voltage (*V*_appl_) was corrected for the built-in voltage (V_bi_) that arises from the WF difference between the contacts. When the applied voltage is greater than *V*_bi_, dark current density throughout all devices scales quadratically with voltage, indicative of SCLC. The *J*^0.5^−*V* curves of the devices based on PEDOT:PSS/PEIE are presented in Fig. 2, and the corresponding electron mobilities are summarized in Supplementary Table S1 online. Among the PEDOT:PSS/PEIE films, those cast from solutions with pH = 9.3 and 9.8 exhibited relatively high electron mobilities of 5.13 × 10^−4^ cm^2^V^−1^S^−1^ and 2.91 × 10^−4^ cm^2^V^−1^S^−1^, respectively. These values are of the same order of magnitude as those of bare ZnO and PEIE (see Supplementary Fig. S4 and Table S1 online).

### Film morphology

**Figure 3:**
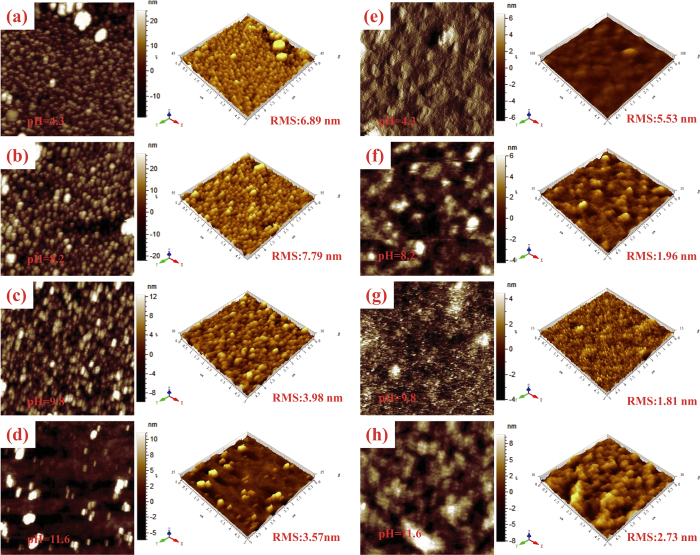
Surface morphology characteristics. Tapping-mode AFM height measurements and three-dimensional images of ITO/PEDOT:PSS/PEIE surfaces prepared from PEIE solutions of (**a**) pH = 4.3, (**b**) pH = 8.2, (**c**) pH = 9.8 and (**d**) pH = 11.6. Tapping-mode AFM height measurements and three-dimensional images of ITO/PEDOT:PSS/PEIE/P3HT:PC_61_BM surfaces prepared from PEIE solutions of (**e**) pH = 4.3, (**f**) pH = 8.2, (**g**) pH = 9.8 and (**h**) pH = 11.6.

The morphologies of the PEIE films with different pH values deposited on PEDOT:PSS were investigated via atomic force microscopy (AFM), and the results are displayed in Fig. 3a–d. The topography and surface roughness of a PEIE film coated on ITO are also provided for comparison in Supplementary Fig. S5 online. The root mean square (RMS) roughness of the bare PEDOT:PSS layer was ~1.03 nm, and the PEIE layer spin coated on ITO was much rougher, with an RMS roughness of ~8.72 nm (see Supplementary Fig. S5 online). As reported in the literature, the 10-nm-thick PEIE layer did not uniformly cover the ITO surface but instead formed PEIE islands (see Supplementary Figs S5 and S6 online), resulting in a rough and inhomogeneous coating. However, the morphology of the PEIE films was strongly influenced by the substrate and by the pH values of the PEIE solutions. When coated on PEDOT:PSS, the thin layers of PEIE with pH values ranging from 4.3 to 11.6 exhibited a much more homogenous morphology with reduced RMS roughness (Fig. 3a–c), and the films with pH values of 9.8 and 11.6 exhibited substantially lower RMS roughnesses of 3.98 and 3.57 nm, respectively, implying improved compatibility between the PEIE and the PEDOT:PSS. For the PEIE film cast from the highly basic solution with a pH of 11.6, a small number of aggregates could be distinctly observed attached to the smooth PEIE film, probably as a result of NaOH domains (Fig. 3d). The difference in the morphologies of PEIE films with different pH values resulted in obvious morphological differences on the upper active layer (Fig. 3e–h). The morphology of the P3HT:PC_61_BM layer coated on the PEDOT:PSS/PEIE film became smoother as the pH value increased from 8.2 to 9.8. By contrast, a P3HT:PC_61_BM surface with an increased RMS roughness of 2.73 nm was clearly observed on the nonuniform PEIE film with a pH of 11.6, which would not be favorable for device performance.

**Figure 4:**
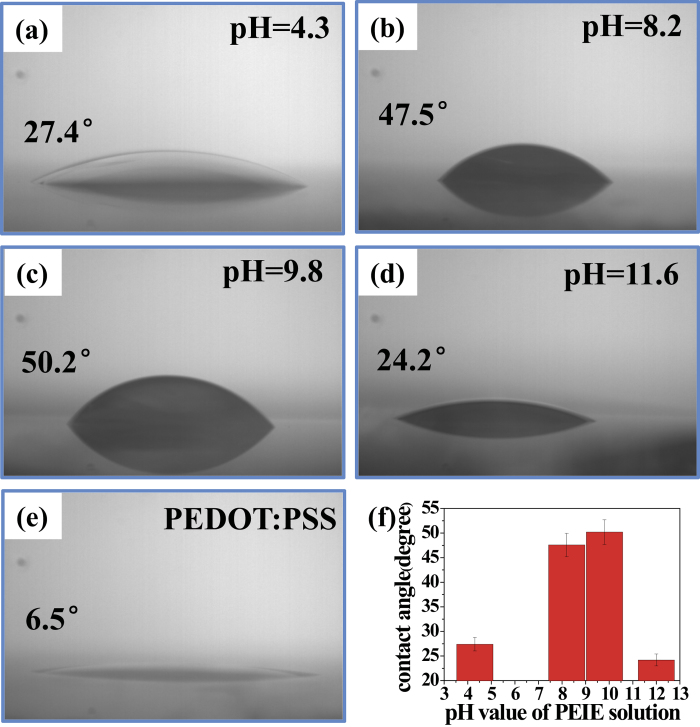
Contact angle measurements. Contact angle measurements indicating the hydrophilicity of PEIE with various pH values on ITO/PEDOT:PSS: (**a**) pH = 4.3, (**b**) pH = 8.2, (**c**) pH = 9.8, (**d**) pH = 11.6 and (**e**) bare ITO/PEDOT:PSS. (**f**) Surface contact angle of ITO/PEDOT:PSS/PEIE as a function of the pH value of the PEIE solution.

Interfacial compatibility is crucial for PSC performance and for the processing of multi-layer devices. Therefore, to evaluate the wettability of the PEDOT:PSS/PEIE films, contact angle measurements were performed, as shown in Fig. 4a–e. Compared with pristine PEDOT:PSS, which exhibited a contact angle of 6.5°, all PEIE-modified PEDOT:PSS films exhibited much higher contact angles, indicating improved hydrophobicity. This substantial change in the wettability of the PEDOT:PSS/PEIE films could facilitate the deposition of a hydrophobic active layer and also improve interfacial contact. Simultaneously, the hydrophobic nature of the PEDOT:PSS/PEIE films could mitigate the hygroscopic nature of the PEDOT:PSS layer and thus protect the device from water damage. Among the modified films, that prepared with PEIE coated from a solution with a pH of 9.8, without any added acid or alkali, exhibited the highest contact angle of 50.2° (Fig. 4c) because both HPF_6_ and NaOH increased the hydrophilicity of the PEIE films.

### Photovoltaic performance and characterization

**Figure 5:**
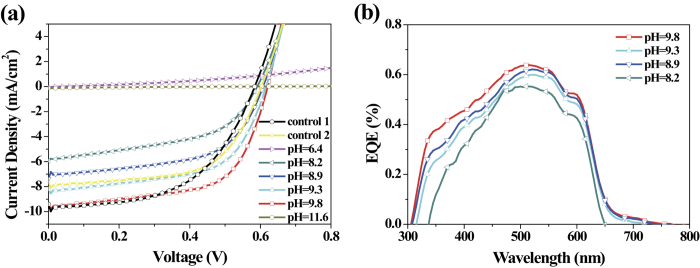
Photovoltaic performance with a P3HT:PC_61_BM-based active layer. (**a**) *J*-*V* characteristics of inverted cells with P3HT:PC_61_BM-based active layers and with various cathode interfacial layers under AM 1.5G irradiation at 100 mW/cm^2^. (**b**) EQE spectra of the inverted cells with PEIE-modified PEDOT:PSS ETLs.

**Figure 6:**
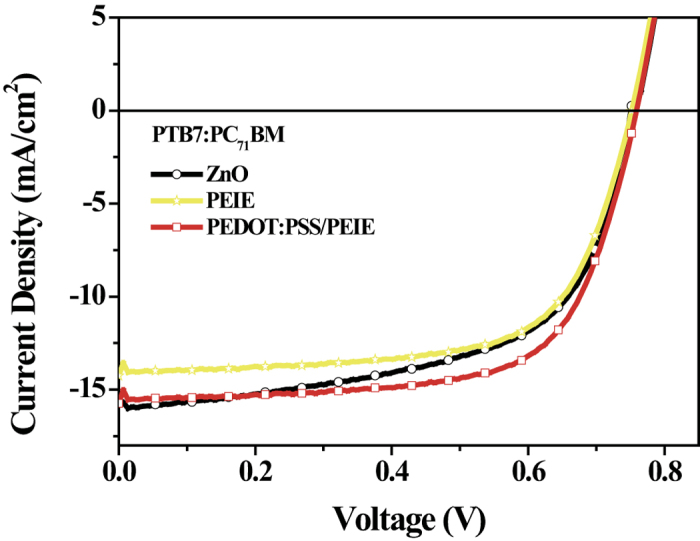
Photovoltaic performance with a PTB7:PC_71_BM-based active layer. *J*-*V* characteristics of inverted cells with PTB7:PC_71_BM-based active layers and with ZnO, PEIE and PEDOT:PSS/PEIE cathode interfacial layers under AM 1.5G irradiation at 100 mW/cm^2^.

The current density-voltage (*J*-*V*) characteristics of inverted polymer cells fabricated with PEDOT:PSS/PEIE as the ETL and with the configuration glass/ITO/PEDOT:PSS/PEIE/P3HT:PC_61_BM (1:1 w/w)/MoO_3_/Ag under AM 1.5G irradiation at 100 mW·cm^−2^ are shown in Fig. 5a, and the electrical parameters are listed in Table 1. Three control devices fabricated with bare PEDOT:PSS, bare PEIE and bare ZnO as their ETLs were also fabricated and measured for comparison. Because of the nature of high-work-function PEDOT:PSS as a hole transport layer, the device fabricated with this material as the ETL did not operate. When PEIE-modified PEDOT:PSS prepared at a high pH value (pH = 4.3 and 6.4) was used as the ETL, the resulting devices demonstrated zero or extremely low PCEs. This is because the excessively high WF (~4.9 eV) created a very high energy barrier for electron injection. As the pH value of the PEIE increased, the PCE and the overall device performance parameters, such as the short-circuit current density (*J*_SC_), the *V*_OC_ and the fill factor (FF), dramatically improved. As anticipated, the device based on PEDOT:PSS/PEIE with a pH of 9.8 achieved the best PCE of 3.64%, with a *J*_*SC*_ of 9.43 mA cm^−2^, a *V*_OC_ of 0.62 V, and an FF of 62.2%. The improved *V*_OC_ is ascribed to the substantially reduced WF of the PEDOT:PSS/PEIE, which allowed for better energy alignment, as supported by the UPS results (Fig. 1c). Moreover, the formation of favorable interfacial dipoles may have enhanced charge extraction and reduced charge recombination, thereby leading to the improvements in *J*_SC_ and FF, coincident with the remarkable reduction in the series resistance (Rs) from 194.2 to 3.6 Ω cm^−2^. Additionally, higher interfacial contact, a more favorable active morphology, stronger light absorption and improved charge mobility contributed to the improvements in *J*_SC_ and FF. The external quantum efficiency (EQE) spectra of the various devices are shown in (Fig. 5b), with the corresponding *J*_SC_ values. As the pH value increased, an increase in the EQE at wavelengths between 300 nm and 750 nm was observed, and the device with the best PCE exhibited the highest EQE (approximately 64%) at a wavelength of 500 nm. Notably, because of its more suitable WF and better wettability with the active layer, the PEDOT:PSS/PEIE layer fabricated at pH = 9.8 exhibited a maximum PCE of 3.64%, even higher than that of bare ZnO (2.98%), thereby illustrating the superior interfacial modification of the PEDOT:PSS/PEIE. When PEIE alone was used as an interfacial layer, the devices yielded a PCE of only 2.84% because PEIE is both insulating and aliphatic and has only a large bandgap; it serves not as an efficient electron injection layer but rather as a surface modifier to reduce the WF of the ITO. However, when the pH of the PEIE was further increased to 11.6, although the photo-induced current was barely detectable, a notable R_s_ of 3946.9 Ω cm^−2^ was measured instead, probably because of the unfavorable morphology of the active layer and the poor interfacial contact resulting from the presence of NaOH aggregates, as revealed by the AFM and contact angle measurements (Fig. 3d,h and 4d). The weak diode quality, along with a very low leakage current, is clearly evident from the dark *J*-*V* curve (see Supplementary Fig. S7 online). Interestingly, the PEDOT:PSS/PEIE films also functioned well in inverted solar cells with a PTB7:PC_71_BM-based active layer, demonstrating the versatility of this novel ETL. Compared with devices fabricated with ZnO and bare PEIE film, the devices with PEDOT:PSS/PEIE interfacial layers achieved a remarkably improved PCE of 7.94%, with a *J*_*SC*_ of 15.74 mA cm^−2^, a *V*_OC_ of 0.76 V, and an FF of 66.4%. The related *J*-*V* characteristics and performance data are provided in Fig. 6 and Table 2.

### Evaluation of cell stability

**Figure 7:**
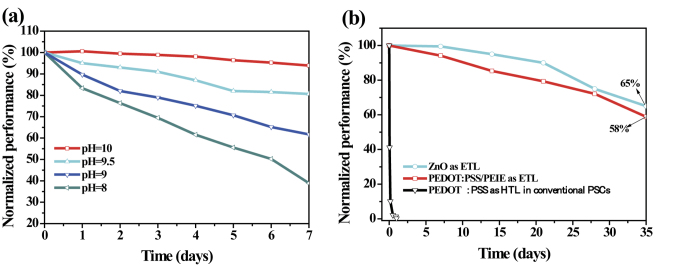
Cell stability measurements. Normalized PCEs of inverted cells fabricated with interfacial layers of (**a**) PEIE-modified PEDOT:PSS with different pH values and (**b**) PEDOT:PSS/PEIE, PEDOT:PSS and ZnO as a function of storage time in air without any encapsulation.

Figure 7 shows the normalized efficiency of the inverted cells fabricated with ETLs of PEDOT:PSS modified with PEIE at different pH values as a function of storage time in air without any encapsulation. In contrast to the sharp drop observed in the PCEs of the devices fabricated with pristine PEDOT:PSS (see Supplementary Fig. S8 online), the PEIE-modified PEDOT:PSS interlayers ensured the long-term stability of the devices. As the pH of the PEIE increased from 8.2 to 9.8, the PCEs of the devices were maintained at between 39% and 94% of their initial efficiency after storage in air for 7 days. This improved stability can be partially ascribed to the reduced hygroscopicity of the PEDOT:PSS covered with PEIE. We further prolonged the storage time of the devices to 35 days and found that the PCE of the inverted cell based on PEDOT:PSS/PEIE with a WF of 4.1 eV (pH = 9.8) maintained 58% of its initial value, indicating device stability comparable to that of bare-ZnO-based devices (65% of the initial value after storage in ambient air for 35 days). The *J*-*V* characteristics of the inverted cell based on PEDOT:PSS/PEIE (pH = 9.8) as a function of storage time in ambient air are shown in Supplementary Fig. S9 online. Similarly, the incorporation of the PEIE-modified PEDOT:PSS also endowed the device based on PTB7:PC_71_BM with good stability, as shown in Supplementary Fig. S10 online.

**Figure 8:**
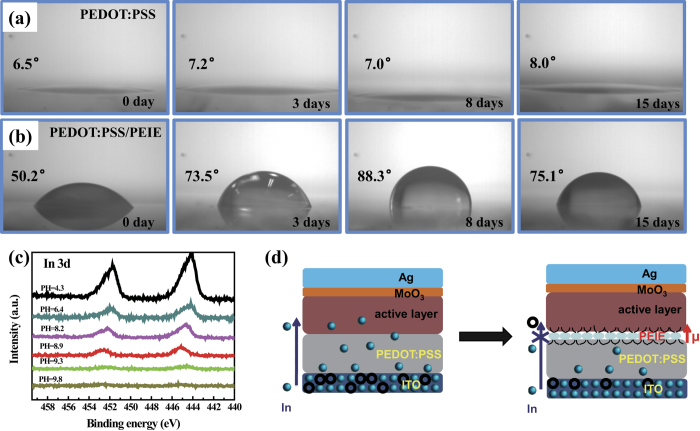
Analysis of cell stability. Contact angle measurements indicating the hydrophilicity of (**a**) PEDOT:PSS films and (**b**) PEDOT:PSS/PEIE films stored in air for 0 days, 3 days, 8 days and 15 days. (**c**) 3d In XPS spectra of PEIE-modified PEDOT:PSS (after storage of the film in air for 15 days). (**d**) Schematic diagram of the stability mechanism of the cell.

To determine the origin of the improved stability of the PEDOT:PSS-based devices, hydrophilicity measurements of PEDOT:PSS and PEDOT:PSS/PEIE films stored in air for 0, 3, 8 and 15 days were performed. The observed change in the hygroscopicity of the PEDOT:PSS films during storage in air is shown in Fig. 8a,b. The contact angle of the pristine PEDOT:PSS film remained below 10° from 0 day to 15 days because of its strong hygroscopicity. However, the contact angle of the PEIE-modified PEDOT:PSS film initially increased from 50.1° to 88.3° and then stabilized at ~70° during storage in air for 15 days. This well-preserved hydrophobicity could protect the PEDOT:PSS from water and oxygen, leading to significantly improved device stability. The contact angles for the PEDOT:PSS films coated with PEIE at the other investigated pH values exhibited similar trends (see Supplementary Fig. S11 online).

Although the reduced hygroscopicity contributed to slower device degradation, there was still a clear difference in the rates of degradation among the cells based on PEDOT:PSS/PEIE prepared at different pH values. Apart from hygroscopicity, the etching of the ITO by the acidic PEDOT:PSS and the subsequent diffusion of In into the active layer is another major cause of device degradation. Therefore, we suspect that the influence of the PEIE on the device stability may also be related to alkalinity of the PEIE film coated onto the PEDOT:PSS, which neutralize the acidity of PSS and thus prevents the diffusion of indium into the active layer. Because the active layer was approximately 150 nm in thickness, which was too thick for In detection, the atomic concentration of In at the interface between the interfacial layer (PEDOT:PSS/PEIE) and the active layer was evaluated. To confirm whether In diffusion was suppressed by the neutralization of the PEDOT:PSS, XPS measurements were performed on the ITO/PEDOT:PSS/PEIE samples (after storage in air for 15 days) were performed to detect the atomic concentration of indium. The In 3d XPS spectra of the PEDOT:PSS films modified with PEIE are presented in Fig. 8c. The PEIE with pH = 4.3 exhibited the strongest In 3d peak, suggesting the highest atomic In concentration in the interfacial layer, which is ascribed to the corrosion of the ITO by the strongly acidic PEDOT:PSS/PEIE. With increasing PEIE alkalinity, the atomic In concentration in PEDOT:PSS significantly decreased. When the pH value of the PEIE was 9.3, free In atoms in the PEDOT:PSS were rare, and with a further increase in the pH to 9.8, the characteristic peak of In 3d could no longer be observed, indicating the absence of In on the surface of the PEDOT:PSS/PEIE. The substantial suppression of the atomic In concentration in the interfacial layer can be attributed to the dramatically reduced acidity of the PEDOT:PSS as a result of neutralization by the alkaline PEIE, which prevented ITO corrosion. As a result, the diffusion of atomic In from the interfacial layer into the active layer was inhibited, and introduction of gap states caused by the In acting as trapping sites for photo-excited carriers could also be avoided, consequently leading to an improved cell lifetime (see the schematic illustration in Fig. 8d).

## Discussion

In conclusion, the use of PEIE-modified PEDOT:PSS films with a low WF of 4.1 eV as ETLs for inverted solar cells was demonstrated. The WF of the PEDOT:PSS film could be well tuned from 4.9 eV to 4.1 eV, and the charge selectivity could be simultaneously altered from supporting hole transport to supporting electron transport, by simply controlling the pH value of the PEIE solution. It was also found that a thin layer of PEIE spin coated onto the PEDOT:PSS surface in an ambient atmosphere could give rise to higher interfacial contact, a more favorable active morphology, stronger light absorption and improved charge mobility. By virtue of these advantageous properties, inverted PSC fabricated with the PEDOT:PSS/PEIE film as ETL achieved a PCE of 7.94%, which is even higher than that of a device with only a ZnO layer. More importantly, the hydrophobic and alkaline PEIE not only reduced the hygroscopicity of the PEDOT:PSS but also neutralized the acidic PEDOT:PSS to avoid the etching of the ITO, remarkably improving the device stability. These results illustrate the potential of PEIE-modified PEDOT:PSS for use as an efficient ETL for commercial printed electronic devices.

## Methods

### Materials

P3HT (Rieke Metals), PTB7 (1-Material), PC_61_BM and PC_71_BM (American Dye Source, Inc., 99.5%) were used as received. Poly(3,4-ethylenedioxythiophene):poly(styrene sulfonate) (PEDOT:PSS) (Clevios PVP AI 4083) was purchased from Heraeus. PEIE (M_w_ = 70000 g/mol, 35–40 wt% in H_2_O) was acquired from Aldrich. The PEIE was further diluted with 2-methoxyethanol to a weight concentration of 0.4%.

### Sample preparation

A PEIE solution (14 ml) was prepared at a weight concentration of 0.4% in 2-methoxyethanol. The resulting solution had a pH value of 9.8. Subsequently, 100 μl of a nonvolatile acid, HPF_6_ (60 wt% in water), was added to the original PEIE solution to obtain a solution with a pH of 4.3. Solutions with pH values of 6.4, 8.2, 8.9, and 9.3 were also prepared in a similar manner by adding smaller volumes of HPF_6_. A solution with a pH of 11.6 was also prepared by adding a piece of solid NaOH (0.19 g) to the original PEIE solution. The pH values were measured using a pHS-25 pH meter (Shanghai Precision & Scientific Instrument Co., Ltd.). The pH meter was calibrated using a standard buffer solution with a pH of 4.00 before use.

### Solar cell fabrication

To fabricate the inverted cells, ITO-coated glass substrates were first cleaned via sequential ultrasonic agitation in acetone, detergent, deionized water, and isopropanol, followed by 15 min of a UV ozone treatment. PEDOT:PSS was spin coated onto clean ITO substrates at a speed of 4000 rpm. The films were then annealed at 140 °C for 20 min. The thickness of each PEDOT:PSS film was approximately 40 nm, as determined using a profilometer (Alpha-Step-IQ). PEIE was spin cast onto the PEDOT:PSS films (for combined PEDOT:PSS and PEIE) from PEIE solutions of different pH values at a speed of 5000 rpm for 1 min and an acceleration of 1000 rpm/s. The films were then annealed at 100 °C for 10 min on a hot plate in ambient air. The approximate thickness of each film was 10 nm. Reference ETLs consisting of bare PEDOT:PSS, PEIE and ZnO were spin coated onto pre-cleaned ITO substrates at speeds of 4000 rpm, 5000 rpm and 4000 rpm, respectively, for 1 min. A 1,2-dichlorobenzene solution of P3HT and PC_61_BM (1:1 w/w, polymer concentration of 10 mg ml^−1^) was then spin coated on top of the dry PEDOT:PSS film at 800 rpm for 30 s. After drying for 2 h, the film was annealed at 150 °C for 10 min in an N_2_-filled glove box. The cell was finished by thermally evaporating MoO_3_ (7 nm) and Ag (100 nm) under 4 × 10^−4^ Pa through a shadow mask to form an active area of ∼4 mm^2^ (determined via microscope). For the PTB7:PC_71_BM-based devices, PTB7:PC_71_BM with a nominal thickness of 180 nm was prepared by spin coating a mixed solution of chlorobenzene/1,8-diiodoctane (97:3% by volume)^6^ (concentration of 25 mg ml^−1^) at 1000 rpm for 2 min. For cells with the conventional configuration (ITO/PEDOT:PSS/P3HT:PC_61_BM/LiF/Al), HTLs of PEDOT:PSS (∼35 nm) were spin coated at 4000 rpm for 1 min onto pre-cleaned ITO substrates. Then, the BHJ active layers, lithium fluoride (LiF) (7 Å) and Al (100 nm), were deposited using the same conditions used for the inverted cells.

### Characterizations

The current-voltage (*J*-*V*) characteristics of the devices were characterized using a Keithley 2400 Sourcemeter. The currents were measured in the dark and under 100 mW·cm^−2^ simulated AM 1.5G irradiation (Abet Solar Simulator Sun 2000). All measurements were performed under the ambient atmosphere at room temperature. The incident photon-to-electron conversion efficiency spectra (IPCE) were detected under monochromatic illumination (Oriel Cornerstone 260 1/4 m monochromator equipped with an Oriel 70613NS QTH lamp), and the calibration of the incident light was performed using a monocrystalline silicon diode. The transmittance spectra were analyzed via UV-vis spectroscopy (Perkin Elmer Lambda 750). The morphologies of the PEDOT:PSS/PEIE films were investigated via atomic force microscopy (AFM) using a Digital Instrumental Nanoscope 31 operating in tapping mode. The thicknesses of all layers were measured using a surface profilometer (Alpha-Step-IQ). XPS studies were performed using a Thermo-VG Scientific ESCALAB 250 photoelectron spectrometer with a monochromatic Al (Kα) (1,486.6 eV) X-ray source. The base pressure in the XPS analysis chamber was 2 × 10^−9^ mbar. For the UPS measurements, the He I (21.22 eV) radiation line from a discharge lamp was used, with an experimental resolution of 0.15 eV. All UPS measurements of the onset of photoemission for determining the WF were performed using standard procedures with a −5 V bias applied to the sample.

### Space-charge-limited current (SCLC) mobility measurement

To characterize the carrier mobilities of the modified devices, electron-only devices were fabricated. These electron-only devices used a diode configuration of ITO/interlayer/P3HT:PC_61_BM/LiF/Al. The carrier mobility was measured using the SCLC model at low voltage^37^, which is described by Equation 1:
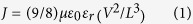
where ε_0_ is the permittivity of free space (8.85 × 10^−12^ F m^−1^), ε_r_ is the dielectric constant of P3HT or PC_61_BM (assumed to be 3), μ is the electron mobility, V is the applied voltage, and L is the film thickness^36^,^38^. The thickness of the BHJ blend for the SCLC measurement was approximately 130 nm. After fitting the results to a space-charge-limited functional form, *J*^0.5^ versus *V* was plotted, as shown in Fig. 2 and Supplementary Fig. S4 online.

## Additional Information

**How to cite this article**: Zhang, Y. *et al.* Low Work-function Poly(3,4-ethylenedioxylenethiophene): Poly(styrene sulfonate) as Electron-transport Layer for High-efficient and Stable Polymer Solar Cells. *Sci. Rep.* **5**, 12839; doi: 10.1038/srep12839 (2015).

## Supplementary Material

Supplementary Information

## Figures and Tables

**Table 1:** Summary of the photovoltaic performances of inverted P3HT:PC_61_BM solar cells with various ETLs.

**Cell**^a^	**ETL**	***J***_**sc**_**(mA cm**^**−2**^)	***V***_**oc**_**(V)**	**FF (%)**	**best PCE (%)**	**average PCE**^b^ **(%)**	**R**_**s**_^c^ **(Ω cm**^**−2**^)	**R**_**sh**_^c^ **(Ω cm**^**−2**^)
control 1	ZnO	9.41	0.58	54.6	2.98	2.80 ± 0.10	2.1	701.9
control 2	bare PEIE	7.64	0.60	61.9	2.84	2.70 ± 0.10	15.6	614.0
control 3	bare PEDOT:PSS	—	—	—	—	—	—	—
A	pH = 4.3	0.00	0.00	∞	0.00	0.00 ± 0.00	-	-
B	pH = 6.4	0.03	0.03	24.8	0.00	0.00 ± 0.00	194.2	2521.7
C	pH = 8.2	5.94	0.59	49.2	1.72	1.50 ± 0.20	40.9	256.6
D	pH = 8.9	7.37	0.60	54.8	2.42	2.20 ± 0.20	12.6	397.9
E	pH = 9.3	8.22	0.61	59.8	3.00	2.80 ± 0.20	4.5	341.9
**F**	**pH = 9.8**	**9.43**	**0.62**	**62.2**	**3.64**	**3.40 ± 0.20**	**3.6**	**333.1**
G	pH = 11.6	0.14	0.57	15.1	0.01	0.01 ± 0.00	3946.9	2299.2

^a^Configuration of control cells 1, 2, and 3: glass/ITO/ETL/P3HT:PC_61_BM (1:1 w/w, 130–180 nm)/MoO_3_(7 nm)/Ag (90 nm). Configuration of cells A, B, C, D, E, and F: glass/ITO/PEDOT:PSS/PEIE/P3HT:PC_61_BM (1:1 w/w, 130–180 nm)/MoO_3_(7 nm)/Ag (90 nm).

^b^All PCE values are averages over 5 cells. The values in bold represent the best-performing device, with the glass/ITO/PEDOT:PSS/PEIE/P3HT:PC_61_BM/MoO_3_/Ag structure.

^c^The parameters of R_s_ and R_sh_ correspond to the best PCE.

**Table 2:** Summary of the photovoltaic performances of inverted PTB7:PC_71_BM solar cells with various ETLs.

**ETL**^a^	***J***_**sc**_**(mA cm**^**−2**^)	***V***_**oc**_**(V)**	**FF (%)**	**best PCE (%)**	**average PCE**^b^ **(%)**	**R**_**s**_^c^ **(Ω cm**^**−2**^)	**R**_**sh**_^c^ **(Ω cm**^**−2**^)
ZnO	15.63	0.75	60.8	7.12	6.90 ± 0.20	9.8	701.9
bare PEIE	14.25	0.75	65.5	7.00	6.80 ± 0.20	9.7	2572.3
PEDOT:PSS/PEIE	15.74	0.76	66.4	7.94	7.80 ± 0.10	9.5	2535.3

^a^Cell configuration: glass/ITO/ETL/PTB7:PC_71_BM (1:1.5 w/w, 130–180 nm)/MoO_3_(7 nm)/Ag (90 nm).

^b^All PCE values are averages over 5 cells.

^c^The parameters of R_s_ and R_sh_ correspond to the best PCE.
