# Supplementary material for: Low Work-function Poly(3,4-ethylenedioxylenethiophene): Poly(styrene sulfonate) as Electron-transport Layer for High-efficient and Stable Polymer Solar Cells
Source: Sci Rep. 2015 Aug 4;5:12839. doi: 10.1038/srep12839 (PMC4523875; doi:10.1038/srep12839)
Supplement: Supplementary Information [file srep12839-s1.doc]

**Supplementary information for**

**Low Work-function Poly(3,4-ethylenedioxylenethiophene): Poly(styrene sulfonate) as Electron-transport Layer for High-efficient and Stable Polymer Solar Cells**

*Yong Zhang, Lie Chen, Xiaotian Hu, Lin Zhang & Yiwang Chen**

Y. Zhang, L. Chen, X. Hu, L. Zhang, Y. Chen

College of Chemistry/Institute of Polymers, Nanchang University, 999 Xuefu Avenue, Nanchang 330031, China

L. Chen, Y. Chen

Jiangxi Provincial Key Laboratory of New Energy Chemistry, Nanchang University, 999 Xuefu Avenue, Nanchang 330031, China

E-mail: ywchen@ncu.edu.cn (Y. Chen)

**Figure S1**  UPS spectra cut-off of surface of pure PEDOT:PSS film and PEDOT:PSS with modification from PEIE with different pH value.

**Figure S2**  N1s response in XPS spectra of PEDOT:PSS with modification from (a) pH=4.3 (b) pH=6.4 (c) pH=8.2 (d) pH=8.9 (e) pH=9.3 (f) pH=9.8 (g) pH=11.6 value of PEIE; (h) ratio of [N]:[N^+^] as a function of pH value of PEIE solution

**Figure S3**  Transmittance of PEDOT:PSS/PEIE film and PEDOT:PSS film.

**Figure S4**  *J*^0.5^-*V* characteristics of electron-only devices with PEIE (blue) and ZnO (red) ETLs.

**Table S1**  Electron mobility of ITO/ETL/P3HT:PC_61_BM/LiF/Al structure device with PEIE, ZnO and different pH PEDOT:PSS/PEIE as buffer layer.

| device | pH=8.2 | pH=8.9 | pH=9.3 | pH=9.8 | PEIE | ZnO |
| --- | --- | --- | --- | --- | --- | --- |
| electron mobility(cm^2^V^-1^S^-1^) | 1.47 × 10^-6^ | 5.71 × 10^-5^ | 5.13 × 10^-4^ | 2.91 × 10^-4^ | 5.49 × 10^-4^ | 2.90 × 10^-4^ |


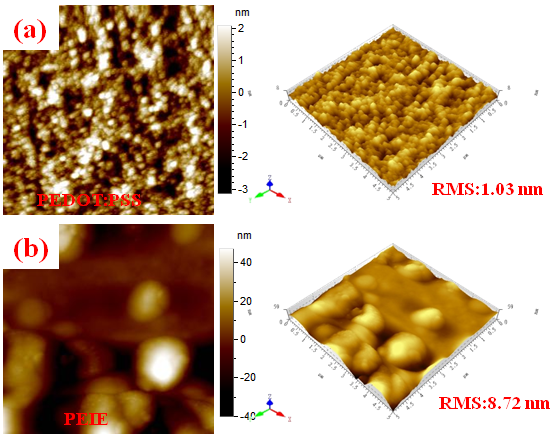


**Figure S5**  Tapping-mode AFM height and three-dimensional images of (a) PEDOT:PSS and (b) PEIE on ITO substrate with an area of 5μm×5μm.


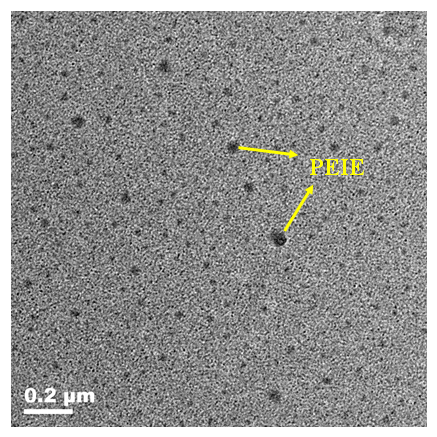


**Figure S6**  Transmission electron microscopy (TEM) image of PEIE.

**Figure S7**  *J*-*V* characteristics of the inverted cells with various interfacial layers (a) ZnO, PEIE, PEDOT:PSS/PEIE (b) PEDOT:PSS/PEIE with different pH in the dark.

**Figure S8**  *J*-*V* characteristics of the conventional cells with PEDOT:PSS as HTL.

**Figure S9**  *J*-*V* characteristics of inverted cell (ITO/PEDOT:PSS/PEIE/P3HT:PC_61_BM/MoO_3_/Ag) without encapsulation for a period of 21-days stored in air.

**Figure S10**  *J*-*V* characteristics of (ITO/PEDOT:PSS/PEIE/PTB7:PC_71_BM/MoO3/Ag) device without encapsulation over a period of 168 h stored in air.


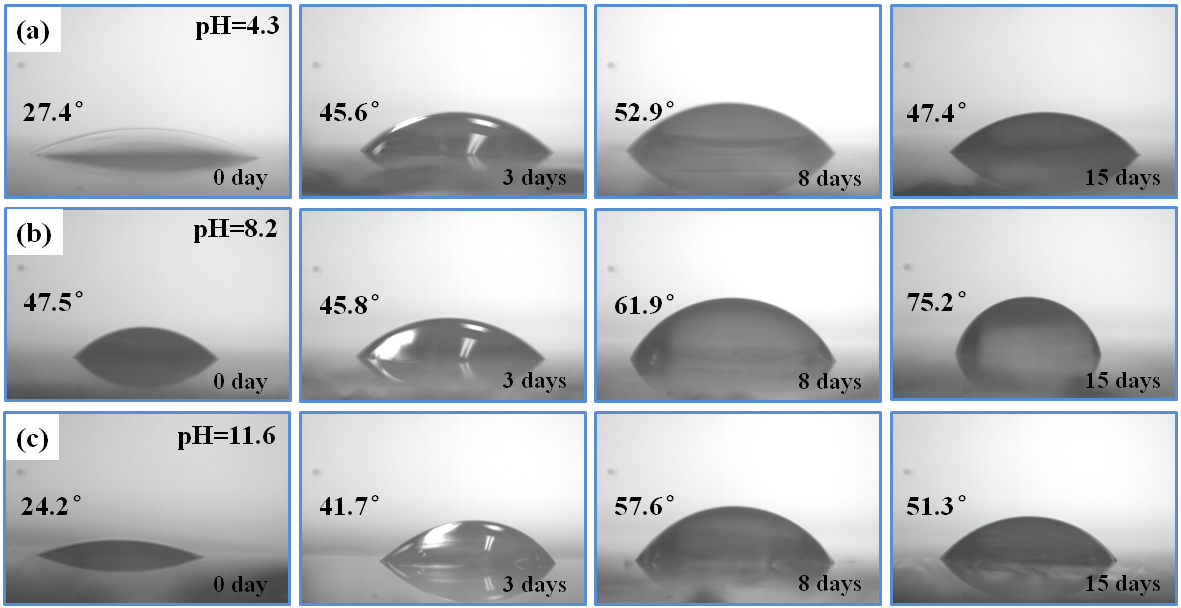


**Figure S11** Contact angle measurements of hydrophilic of (a) PEDOT:PSS film and (b) PEDOT:PSS/PEIE film storage in air for 0 day, 3 days, 8 days and 15days.
